# Medical and Philosophical Intelligence

**Published:** 1815-11

**Authors:** 


### MEDICAL AND PHILOSOPHICAL INTELLIGENCE.

*To the Editor of the London Medical and Physical Journal.*

SIR,

ANY measures adapted to enforce a master in the due fulfilling of his engagements, surely cannot be construed as injurious or disrespectful, but as doing justice to oneself and to the public. On this head, I solicit, through the medium of your Journal, the aid of your superior judgment in remedying, if possible, the present evil, which so much retards our future progress. Many valuable hints have already been suggested on the necessity of medical reform; and it is for the happiness of this country that many important measures are about to be adopted that will greatly benefit the public: there is, however, much still to be enforced before the practice and study of medicine can arrive at perfection.

I, who am an apprentice, embraced a covenant, two years since, with an ardour that gave confidence to myself, and, I believe, to my master. I had then the most flattering hope apparent upon a situation that evinced every possible advantage. But what have those two years shewn me, in the place where I set off, and am likely to continue five years more, behind a counter, dispensing medicines?—is this the sole employment of a young man apprenticed to a surgeon-apothecary and man-midwife? This certainly deserves a consideration in the medical reform:—it is not my unfortunate lot alone, but the lot of hundreds—the practice is become as general as it is vile—it is become a common practice of masters towards their apprentices—few are there whose consciences will allow them to deny it, and still fewer are those who can boast of contrary treatment.

There are a few practitioners who have acquired a degree of excellence; but how many are there who are a disgrace to the profession, and can we wonder at it, when those very measures are entirely neglected which alone could prove the means of remedying it. Thus during our apprenticeship we are duped of acquiring any knowledge of our profession, a profession which requires so much, and so long a study, and which would receive such incalculable benefit were we made masters of its rudiments prior to entering on a course of lectures in the metropolis, the necessary occupation of every medical man.

I remain,

Your obedient and humble servant,

*Sept.* 28, 1815. *An Apprentice in Salisbury.*

Our young correspondent will excuse the freedom with which we have shortened his favour. Though it will be seen that our opinion is not exactly coincident with his, yet we are thankful for his communication, and much pleased to see him bring forward his grievances, rather than murmur in secret. The subject is one which, which for its importance, and from the partial manner in which it is often viewed, we have long wished should be brought before the public.

In the first place, we are not quite satisfied with the apology for the hurry in which the letter is written. Surely, early in the morning, or whilst the master is visiting, time might have been found for a more correct and better-digested letter, from one who shows sufficient proofs of a liberal education. If the hurry is so great, we can promise our correspondent that before the expiration of his remaining five years, his master will be under the necessity of sending him to visit patients, as the practice of midwifery will often confine the former to a spot distant from a sudden call.

But in the mean while, some advice seems necessary to satisfy the impatience, however laudable, of juvenile ardour. Let it be recollected that, till long after the second year of an apprenticeship, few patients will admit the visits of a young man. All that he can expect, is to partake of the attendance of the poor-house, or of the parish; and this he ought not to expect without the assistance of his master. For the few first years of his apprenticeship, then, if he has been articled early in life, he cannot employ his leisure so well as in keeping up his classical acquirements. Though these may be considered unnecessary for his branch of the profession, yet they are extremely useful; and, as every one should look forward towards the highest ranks, he will find the want of such acquirements an insuperable bar. We offer this advice, because a knowledge of languages is with great difficulty, if ever, acquired by men much engaged in business. French should, therefore, come in aid, if leisure permits. Besides these, there are medical books which most practitioners possess, and which, though not completely understood without some knowledge of anatomy, are nevertheless read with advantage, because they furnish facts which, by the interest they excite at that age, are readily brought to recollection in after life. All the commentaries of Van Swieten are of this kind;—the transactions, also, of different societies, the medical periodical publications, and whatever else the library to which a youth may have access furnishes. The writings of Mr. Hunter are irksome to young readers, from the closeness of his reasoning, and because his opinions are so rarely illustrated by cases. On this account, the Commentaries on the Venereal Disease are particularly serviceable, as well as the "Morbid Poisons;" but the latter require leisure and close thinking, and are better understood by those who have made some progress, or commence their studies later in life. Unfortunately, there is no good text-book for a medical tyro, a circumstance which has given encouragement to so many *vade-mecums*, synopses, complete practices, and showy, but fallacious, articles in cyclopædias and dictionaries. All these should be read with the greatest caution. They promise a facility at acquiring medical knowledge, which is dangerous as it is fallacious, and also as it indulges, if it does not encourage, indolence.

dolence. Even the first part of Cullen's First Lines, valuable as that work for the most part is, cannot be considered free from that danger, as the present practice in fevers amply proves. To conclude, we trust our young correspondent, on an impartial examination of himself, will find enough to do, without expecting to visit families who, he may depend upon it, will receive him more coolly than can be agreeable to feelings like his.

---

Wednesday the 18th, being St. Luke's Day, the *College of Physicians* held their annual meeting for the Harveian Oration. Dr. MATON was orator, and acquitted himself with much credit. It was thought by some, that his fondness for natural history induced him to dwell too long on the names of some of the early collectors; but this did not prevent his doing ample justice to the modern ones. The whole composition was elegant, as well as pure in its Latinity, and contained as much novelty as could be expected on subjects so often repeated. The only demise which occurred during the year being a character (Dr. Satterley) which had never appeared in print, gave but little scope for an encomium: that little was, however, very judiciously managed. Of the fellows high in the list, very few were present; but the visitors at the oration and the dinner were numerous and respectable.

The following names are become extinct during the year:—

*Fellow.*—Dr. Satterley.
*Licentiate.*—Dr. Domeir.
*Licentiate in Midwifery.*—Dr. Clarke.
*Extra-Licentiate.*—Dr. Luke, of Exeter.

The following are the additions in the new list:—

*Licentiates.*

Dr. William Back.
Dr. Charles Ferguson Forbes, Argyle-street.
Dr. Thomas Donahoo.
Dr. Theodore Gordon, York Hospital.
Dr. Stephen Luke, York-street, Mary-le-bone.
Sir James M'Gregor, Sloane-street, Chelsea.
Dr. David Plenderleath, Reading.
Dr. Robert Richardson, Rathbone-place.
Dr. Robert Chisholm, Hammersmith.
Dr. Miguel Caetano de Castro.
Dr. Hugh Bone.

*Commissioners appointed under the "Act for Regulating Mad-Houses."*

| | |
|---|---|
| Dr. Latham. | Dr. Nevinson. |
| Dr. Hervey. | Dr. Warren. |
| Dr. Haworth. | |

*Secretary to the Commissioners.*—Dr. Richard Powell.

*Censors.*

*Censors.*

| | |
|---|---|
| Henry Halford. | Joseph Ager. |
| William Lambe. | Joseph Cope. |

*Registrar.*—Dr. Clement Hue.

The expectation entertained of building a new College in a more convenient part of the town, induces a backwardness in all temporary repairs, which diffuses an universal gloom, as well as chilliness, in the theatre.

---

Professor Braconnot has been very diligent in making experiments on the nature of fat in animal bodies. Every thing relative to animal chemistry may prove interesting; but for subjects of this kind one accessible record is sufficient. We shall offer a short extract of the whole, referring those who may wish to try the experiments to the Annals of Philosophy for the minuter parts.

"Until the present day, chemists have regarded the fat of organized bodies as the same in composition, possessing the same essential properties, and differing only in the greater or less degree of its consistence; whence the denominations, suet, lard, marrow, fat, &c. admitted by the ancients. The consistence of fat, indeed, varies in a remarkable manner; thus it is hard in the ruminating quadrupeds; softer in man than in graminivorous animals, and almost liquid in the mammiferous amphibiæ, and all the carnivorous animals, whether birds, reptiles, fishes, or insects. The consistence of fat varies not only in different kinds of animals, but also according to the part of an animal in which it is situated; the age, the sex, and the physical constitution of the individual. It is very hard in the vicinity of the kidneys, softer in the epiplon, the mesentery, the intestines, and around the other moveable viscera; and almost fluid where it lines the orbit. Peculiar accidental circumstances alter also the consistence of fat, as is found in steatomatous tumours, in old hernias of the epiplon, and in some sebaceous tumours, which sometimes have the hardness of a calculus.

"Reflecting upon this infinite variation in the consistence of fat, and observing besides that suet and oil form the two extremes, I was led to imagine that these two bodies, mixed together in different proportions, might produce the varieties of fat found in organized bodies. With this view, I endeavoured to discover some chemical re-agent that would separate the suet and the oil, which I supposed existed in all fat bodies; I could not, however, find one fitted for the purpose, but a very simple method suggested itself to my mind, and fully confirmed my conjecture. It depends on the physical property of bibulous paper for imbibing oil, while it is not at all affected by suet in its pure state. By applying this method of analysis to fat bodies, I was enabled to discover the two substances of which they are composed, and to determine their respective proportions."

Prof. B. found that one hundred parts of melted butter, of a good

 quality, quality, made during summer, yielded as a product, at the temperature of zero, oil 60—suet 40.

But these proportions are liable to variation, according to the physical constitution of the cow, the nature of her food, and the place where she is domiciled. Thus one hundred parts of winter butter from the Vosges yielded, at the temperature of zero, oil 35—suet 65.

One hundred parts of hog's lard at zero of the thermometer furnished, as constituent principles, oil 62—suet 38.

When well washed and freed from its vascular envelopes by fusion, beef marrow had a firm consistence at the temperature of 2° R.; in this state it was pressed in gray paper until it ceased to stain it, and he obtained, suet 76—oil 24.

One hundred parts of mutton marrow, washed and melted, gave, at the temperature of 2° Reaumur, suet 26—oil 74.

One hundred parts of olive oil, at the temperature of 5° R., produced, oil of a greenish yellow 72—very white suet 28.

These experiments just detailed on the *fat principles*, authorise us to think that all the rest are similarly composed of fluid oil and a solid substance, which we find even in the oils which have the least dispostion to freeze, as recent linseed oil, which deposits during a cold night very regular conical crystals.

On examining, with the naked eye, suet, as we meet with it in animals, that is, that which envelopes the kidneys in the ruminating quadrupeds, we find it arranged in conchoid, square, or orbicular masses, separated from each other by membranous laminæ. If we examine each of these masses attentively, we see them entirely formed of an innumerable multitude of oblong globules, transparent and brilliant like a crystallized salt, and which seem tied to each other by a very loose membranous texture, like the grains of a starch in a boiled potatoe. We may separate these globules from each other by slightly macerating the suet in cold water, and shaking the whole over a hair sieve. We then obtain a powder which resembles starch when it has been dried on gray paper. These membranous vesicles containing the suet, did not appear to have the same structure as the cellular texture, which is formed, as we know, by the junction and mutual adherence of the cellules which communicate with each other.

---

The last number of the Repository contains a query whether the administration of sulphuric acid to a wet nurse has been attended with pernicious consequeuces to the infant. Mr. Bartley produces two instances in which children were seized with convulsions under such circumstances. It does not appear that any chemical experiments were made to ascertain whether any or what change had been made in the milk. It is certain that some substances swallowed may be detected, as in the butter when cows are fed on turnips, or meet with albaceous plants. The inquiry is particularly important, as a part of animal chemistry in which the organs themselves are concerned.

*Extract*

The case of Hydrophobia cured by bleeding rests on the authority of Dr. HUFFELAND. We are not yet favoured with the particulars.

---

*Extract from M. Klopstock's Travels on Mount Caucasus.*—Though we were obliged to use all possible dispatch in order to reach Stephan-Tzminda before nightfall, yet we were detained longer than we wished at Tschim by an unpleasant accident. One of my pack-horses fell ill, and grew quite furious. He kicked out with his hind legs, stamped upon the ground, and ran against the walls. He trembled in every joint, and was bathed in sweat. He staled much, the urine being of a dark yellow colour, and frequently broke wind. His respiration, however, was unobstructed; and the Ossites told me his disorder was occasioned by a kind of poisonous plant, which had been very frequent among the hay the preceding year. To prevent, therefore, any inflammation of the stomach, the Cossacks gave him milk and gunpowder, and took some blood from him. These precautions proved unavailing, for in about an hour the animal died. On opening him, no symptoms of inflammation was discovered, either in the chest or abdomen. The stomach was crammed with hay which he had eaten; but the intestines were empty, and distended with wind. The Tartars call this disease *horse-death.*

---

*Description of a Calculus found in a Tumour in the left Hypochondrium.*—M. PENADA, an Italian surgeon, has published, in the Memoirs of the Italian Society of Sciences, the following:—"The calculus existed in a female fifty years of age, and came out through an ulcer which was formed in the tumour. It was precisely of the shape of a hen's egg: the colour was dark grey, and its surface granulated and shagreened. On removing the external coating, the calculus presented a lamellated structure and a bright yellow colour: its consistency was compact, but not very hard; and it weighed in all one ounce two drachms. When thrown into water before opening it, it floated for a few seconds, and then fell to the bottom of the liquid, giving out some air bubbles.

"It was cut into two parts parallel to its axis: interiorly it presented from ten to twelve concentric layers, which decreased gradually in thickness, analogous to walnut-tree wood. These layers were separated by a black streak around a circle of a deep yellow, which diminished in breadth as it approached the centre, and the surface of which was as brilliant as marble or highly polished wood. In the centre of these ellipses a perfectly spherical body was remarked, of a fine white, formed of a substance less compact than the rest of the calculus; of a crystalline scaly texture, and having nearly the form and consistence of the crystalline of a bull's-eye coagulated by heat. This nucleus was surrounded by a black circle more deeply coloured than the ellipses just mentioned."

On the chemical analysis of this calculus, Professor Melandri, of Padua, obtained the following results:

"The central substance, or white nucleus, was entirely soluble in ether, crystallizable and combustible; when thrown upon red-hot coals, it emitted the smell of incense. The cortical part was only partially soluble in alcohol and ether. The soluble part crystallized, and burned with a white flame and a smell of wax. The insoluble part burned with the smell of animal matter. Professor Marabelli concludes from these experiments, that the calculus was formed entirely of adipocire modified by a resinous principle, and the cortical part of adipocire very pure, and of an animal substance."

This was probably an incisted tumour of adipocere, the external tunic of which had begun to assume a calculous form.

---

We lament to have to announce the death of GEHLEN, many years the editor of an excellent Journal on Chemistry and other Sciences, and himself an eminent chemist. He fell a victim to his ardent desire to promote the advancement of chemical knowledge. He was preparing, in company with his colleague Mr. Rahland, some *arseniated hydrogene gas;* and, whilst watching for the full developement of this air from its acid solution, trying at every moment to judge from its particular smell when that operation would be completed, he inhaled the fatal poison, which has robbed science of his valuable services.

---

### LECTURES.

The Diseases of the present month having varied but little from the preceding, we shall, on account of our great press of matter, defer our remark on particular cases to the next month.

### METEOROLOGICAL REGISTER.

*From September the 25th, to October the 25th,* 1813.

Kept by C. BLUNT, Philosophical Instrument Maker, No. 38, Tavistock-Street, Covent-Garden.

| Moon. | Day. | Wind. | Barometrical Pressure. | | | Temperature. | | | |
|---|---|---|---|---|---|---|---|---|---|
| | | | Max. | Min. | Mean. | Max. | Min. | Mean. | |
| ☽ | 26 | S | 29·84 | 29·80 | 29·812 | 70 | 44 | 59·75 | Rain during the Night |
| | 27 | S | 30·11 | 29·94 | 29·997 | 72 | 38 | 58 5 | Rain |
| | 28 | S | 30·15 | 29·99 | 30·062 | 68 | 44 | 55·75 | Fair |
| | 29 | SW | 29·54 | 29·41 | 29·427 | 67 | 45 | 57·75 | Rain A. M. |
| | 30 | SW | 29 66 | 29·64 | 29·65 | 66 | 43 | 57·5 | Rain at Night |
| | 1 | W | 29·82 | 29·82 | 29·82 | 66 | 42 | 57·75 | Rain |
| ● | 2 | W | 30·12 | 29 90 | 29·932 | 65 | 41 | 57·25 | Rain |
| | 3 | SW | 30 20 | 30·13 | 30·157 | 62 | 41 | 55·75 | Rain |
| | 4 | S | 30·11 | 30 04 | 30·075 | 63 | 40 | 55 75 | Rain |
| | 5 | S | 30·06 | 29·99 | 30·013 | 60 | 38 | 52· | Rain |
| | 6 | S | 30·07 | 29 95 | 30·00 | 60 | 40 | 51·75 | Rain |
| | 7 | E | 30·30 | 30·22 | 30·217 | 58 | 42 | 51·5 | Rain |
| | 8 | W | 30·20 | 30·20 | 30·20 | 57 | 43 | 51·75 | Rain |
| | 9 | SW | 30·06 | 30·03 | 30·042 | 55 | 42 | 49·5 | Rain |
| ◐ | 10 | E | 30·03 | 29 83 | 29·935 | 54 | 41 | 51· | Rain |
| | 11 | E | 29·80 | 29·68 | 29·735 | 53 | 40 | 48·5 | Rain |
| | 12 | E | 29·74 | 29·71 | 29·722 | 53 | 35 | 46·75 | Rain |
| | 13 | SE | 29·77 | 29·74 | 29·755 | 54 | 35 | 46·75 | Fair |
| | 14 | SW | 29 74 | 29·72 | 29·735 | 58 | 40 | 49·25 | Rain |
| | 15 | S | 29·78 | 29·78 | 29·78 | 60 | 41 | 53·75 | Fair |
| | 16 | S | 29·84 | 29·80 | 29·827 | 65 | 45 | 53 5 | Fair |
| | 17 | S | 29·86 | 29·83 | 29 887 | 63 | 36 | 51·25 | Fair |
| ○ | 18 | S | 30· | 29 63 | 29·827 | 64 | 41 | 50·5 | Rain |
| | 19 | SW | 29·56 | 29·43 | 29·467 | 66 | 45 | 53·5 | Rain |
| | 20 | S | 29·50 | 29·40 | 29·46 | 66 | 45 | 53·75 | Rain |
| | 21 | S | 29·75 | 29·53 | 29·615 | 67 | 35 | 53· | Fair |
| | 22 | SW | 29·66 | 29 66 | 29·66 | 66 | 36 | 52· | Fair |
| | 23 | SW | 29·61 | 29·54 | 29·577 | 67 | 45 | 54·75 | Rain |
| | 24 | SE | 29·53 | 29·50 | 29·513 | 66 | 41 | 52·5 | Rain |
| ◑ | 25 | SW | 29 57 | 29·54 | 29·555 | 60 | 41 | 51.25 | Fair |

RESULTS.

| | | | | | |
|---|---|---|---|---|---|
| Mean barometrical pressure of the month | | 29·7538 | Mean temperature of the month | | 53·7094 deg. |
| Maximum 30 3, | wind at | E | Maximum 72, | wind at | S |
| Minimum 29·41, | ——— | SW | Minimum 35, | ——— | E |

*Scale exhibiting the prevailing Winds during the Month.*

| N | NE | E | SE | S | SW | W | NW |
|---|---|---|---|---|---|---|---|
| 0 | 0 | 4 | 2 | 12 | 9 | 3 | 0 |

| | Mean barometrical pressure. | Mean temperature. |
|---|---|---|
| From the last quarter on the 26th Sept. to the new moon on the 2d of Oct. | 29·794 | 57·833 |
| ——— new moon on the 2d, to the first quarter on the 10th | 30·135 | 53·156 |
| ——— first quarter on the 10th, to the full moon on the 18th | 29·797 | 52·594 |
| From the full moon on the 18th, to the last quarter on the 25th | 29·588 | 53·714 |

